# Detecting Disease Specific Pathway Substructures through an Integrated Systems Biology Approach

**DOI:** 10.3390/ncrna3020020

**Published:** 2017-04-19

**Authors:** Salvatore Alaimo, Gioacchino Paolo Marceca, Alfredo Ferro, Alfredo Pulvirenti

**Affiliations:** Bioinformatics Unit, Department of Clinical and Experimental Medicine, University of Catania, c/o Dipartimento di Matematica e Informatica, Viale A. Doria 6, 95125 Catania, Italy; alaimos@dmi.unict.it (S.A.); gioacchinopaolomarceca@gmail.com (G.P.M.); ferro@dmi.unict.it (A.F.)

**Keywords:** pathway perturbation, subpathway analysis, microRNAs, drugs, functional analysis.

## Abstract

In the era of network medicine, pathway analysis methods play a central role in the prediction of phenotype from high throughput experiments. In this paper, we present a network-based systems biology approach capable of extracting disease-perturbed subpathways within pathway networks in connection with expression data taken from The Cancer Genome Atlas (TCGA). Our system extends pathways with missing regulatory elements, such as microRNAs, and their interactions with genes. The framework enables the extraction, visualization, and analysis of statistically significant disease-specific subpathways through an easy to use web interface. Our analysis shows that the methodology is able to fill the gap in current techniques, allowing a more comprehensive analysis of the phenomena underlying disease states.

## 1. Introduction

Knowledge base-driven pathway analysis [1] has become a popular approach in complex disease study in order to improve biological insights for a deeper understanding of the molecular mechanisms underlying specific phenotypes.

“Omics” technologies are capable of identifying differentially expressed genes and metabolites associated with specific diseases. On the other hand, biological pathway databases, such as Kyoto Encyclopedia of Genes and Genomes (KEGG) [2,3,4] or Pathway Commons [5], enable the study and the understanding of the system level effects of differential expressions.

Several applications have been implemented to visualize and analyze “Omics” data in the context of known biological pathways [1]. To this purpose, several statistical tests in connection with known biological databases have been used to detect significant pathways. However, the gap between current analysis techniques and the ability to obtain accurate knowledge is broad. Therefore, using such information to better understand the underlying biological phenomena remains a challenge.

Pathway-based analysis techniques can be grouped into three generations of approaches [1]: (i) Over-Representation Analysis (ORA); (ii) Functional Class Scoring (FCS); and (iii) Pathway Topology-based (PT).

First-generation methods statistically evaluate the number of altered genes in a pathway with respect to the set of all analyzed genes [6,7,8,9,10,11,12] and microRNAs (miRNAs) [13]. This approach, however, presents several limitations mainly due to the type, quality, and structure of the annotations available.

Second-generation methods tried to compensate some disadvantages of ORA approaches [14,15]. They consider the distribution of pathway genes in the entire list of genes and perform a FCS, allowing adjustments for gene correlations. However, they do not take into account the magnitude of the gene deregulation when estimating pathway activity. A further limitation lies in the fact that each functional category is analyzed independently without a unifying analysis at a pathway or system level.

The third generation class, Pathway Topology-based methods, can be divided into two subgroups of tools. The first one examines the effect of a phenotype at the global level. The second, and more recent approach, extracts subpathways specifically related to the phenotype under study.

In Draghici et al. [16], an analytical technique based on an impact factor (IF) calculation was introduced. The impact factor is a pathway-level score that takes into account the magnitude of change in genes expression, the type of interactions between genes, and their location in the pathway. Such impact analysis represents an important attempt to allow a deeper level of statistical analysis, informed by more pathway-specific biology. Authors model each pathway as a graph in which nodes represent genes, while edges are interactions between them. Authors also define a gene-level statistic (called perturbation factor, PF) as a linear function of the change in gene expression and the perturbation of its neighborhood. Such a statistic is then combined for each element in a pathway, and a *p*-value is computed by means of exponential distribution.

The analysis presented by Draghici et al. [16] has been further improved by Signaling Pathway Impact Analysis (SPIA) [17], which attenuates the dominant effect caused by the change in expression within PF computation, while reducing the high rate of false positives when the input list of genes is small. SPIA uses a bootstrap procedure to evaluate the significance of the observed perturbation in the pathway. This is combined with a *p*-value computed in ORA style to make a full assessment of the statistical significance of the perturbation of each pathway.

To reduce the number of false positives, Vaske et al. [18] presented PAthway Representation and Analysis by Direct Reference on Graphical Models (PARADIGM), which has been further improved in [19]. PARADIGM is a method to infer patient-specific genetic activity by incorporating information regarding interactions between genes provided in a pathway. The method predicts the degree of alteration in the activity of a pathway by employing a probabilistic inference algorithm. The authors show that their model yields significantly more reliable results than SPIA.

However, both SPIA and PARADIGM completely ignore post-transcriptional regulatory interactions involving miRNAs. To overcome this limitation, Calura et al. [20] developed a new approach, Micrographite, which is able to integrate pathways with predicted and validated miRNA-target interactions. The method, by performing a topological analysis based on expression profiles, is able to identify significant gene circuits specific to a phenotype. The main advantage of the methodology is the ability to accurately describe the cellular context that led to the expression data in input.

Recently, Alaimo et al. [21] proposed Mirna enrIched paTHway Impact anaLysis (MITHrIL), a technique that extends Draghici et al. [16] and Tarca et al. [17], by combining their effectiveness while improving the reliability of the results. The strength of MITHrIL lies in the annotation of pathways with information regarding miRNAs. Starting from expression values of genes and/or microRNAs, MITHrIL returns a list of pathways sorted according to the degree of their deregulation, together with the corresponding statistical significance (*p*-values), as well as a predicted degree of alteration for each endpoint (a pathway node whose alteration, based on current knowledge, affects the phenotype in some way).

The second subgroup of pathway perturbation methods focus on subpathways (i.e., local areas of the entire biological pathway), which have the potential to represent the underlying biological phenomena more accurately. The central idea in shifting the efforts toward subpathways is that when a subpathway responds to a specific condition (e.g., drugs), its corresponding pathway should also respond. The high redundancy of pathways together with their high degree of crosstalk suggests that subpathways respond more effectively and sensitively than the whole pathway. Indeed, key subpathway regions are more relevant for interpreting the associated biological phenomena than whole-pathway approaches. In addition, the cross-talk among subpathways could allow going beyond existing pathway knowledge. Furthermore, perturbations in subpathway regions of metabolic pathways could allow shedding light on understanding the causes of diseases [22,23].

In Li et al. [24], authors introduced Subpathway-GM, which uses a structural node similarity within pathways to identify metabolic subpathways based on information from genes and metabolites. Subpathway-GM has been implemented as a freely available web-based and *R*-based tool. Once the user provides genes and metabolites of interest, the system identifies metabolic subpathways by: (i) mapping genes and metabolites of interest to graphs of pathways after graph-based reconstruction of metabolic pathways; (ii) locating subpathways within pathways according to signature nodes; (iii) evaluating the statistical significance of each subpathways using an hypergeometric test. Subpathway-GM improves the identifications of subpathways with respect to previous methodologies, by taking into account that, from a biological perspective, dysfunctional genes should be closely related to dysfunctional metabolites in pathways.

In Judeh et al. [25], authors introduced Topology Enrichment Analysis frameworK (TEAK), a topology enrichment analysis tool based on Bayesian Networks, to extract linear and non-linear subpathways. TEAK fully accounts for the topological information of subpathways and provides an interactive view of the data in the KEGG pathways. Compared with previous approaches, TEAK does not use differential gene expression analysis to determine modules of interest, and, thus, it is not sensitive to threshold values.

In [26], authors propose DE subs to identify differentially expressed subpathways using RNA-seq data. DEsubs is available as an *R* package. The tool enables users to perform customized analysis on the problem under investigation through several operation modes to uncover local perturbations within networks.

Subpathway-GMir [27] uses a list of condition-specific genes, miRNAs and pathway topologies, and it has been implemented as a freely available *R* package. It aims to identify abnormal metabolic subpathways mediated by miRNAs. The pipeline contains three main components: (i) creation of reconstructed KEGG metabolic pathway graphs (RMPGs) that integrate miRNA-target interactions (supported by low-throughput experiments), by converting KEGG metabolic pathways into graphs with genes as nodes; (ii) mapping of condition-specific genes and miRNAs into RMPGs and identification of miRNA-mediated metabolic subpathways; (iii) evaluation of the significance of candidate subpathways using the hypergeometric test. Subpathway-GMir provides a platform for identifying abnormal metabolic subpathways mediated by miRNAs, and may help to clarify the roles of miRNAs in diseases. The system has been tested also with The Cancer Genome Atlas (TCGA) data.

More recently, [28] proposed time-vaRying enriCHment integrOmics Subpathway aNalysis tOol (CHRONOS) [28], which for the first time introduces a temporal subpathway search built on top of mRNA and miRNA expression data.

Although the literature is rich with useful tools capable of extracting subpathways from expression data, a simple interface, connected with updated sources, is needed. Moreover, the addition of miRNAs in the subpathway extraction technique is essential since many alterations of these post-trascriptional regulators have been found in several cancer types.

In this paper, we propose SPECifIC (SubPathway ExtraCtor and enrICher), a technique for the extraction, visualization, and enrichment of substructures obtained from a meta-pathway built by combining KEGG pathways. The methodology extends MITHRIL [21] to allow efficient nodes perturbation and statistical significance computation. Therefore, starting from expression data of pathological samples and, optionally, a list of Nodes of Interest (NoIs), the method is able to extract statistically significant disease-specific subpathways and perform functional enrichment, allowing further evaluations. The methodology is implemented as a web application, and is available at [29].

## 2. Results

Comparing subpathway extraction and enrichment methods is a complex process. Therefore, we focused on a case study based on two cancer types from TCGA: BRCA (Breast invasive carcinoma) and COAD (Colon adenocarcinoma).

Breast cancer is the most common malignancy in women in several countries [30]. It is a heterogeneous disease, classified into several sub-types, each one involving a different gene set controlling for diverse processes. The identification of cancer sub-type relies on the expression of the estrogen receptor (ER), progesterone receptor (PR), epidermal growth factor receptor 2 (ERBB2), and cytokeratin (CK) protein [31].

Accordingly, three basic therapeutic groups [32] have been identified: (i) the ER positive group, also named “luminal group” and, further divided into luminal A and luminal B, is the most numerous and diverse in terms of gene expression and mutation spectrum; (ii) the HER2 (also called erb-b2 receptor tyrosine kinase 2, ERBB2) amplified group; (iii) the triple-negative breast cancers (TNBCs), also known as “basal-like” breast cancers, characterized by lacking expression of ER, PR and HER2, more frequent in patients with germline BRCA1 mutations.

Colon and rectal cancer, which we refer to as colorectal carcinoma (CRC), are major causes of cancer-associated morbidity and mortality all over the world [33]. Although colon (COAD) and rectal (READ) adenocarcinomas present differences from an epidemiological point of view [34], their molecular characterizations in a non-hypermutated state revealed that the overall patterns of changes in copy number, mRNA and miRNA are each indistinguishable from the other [35]. Accordingly, the application of a cluster analysis method termed “cluster-of-cluster assignments” (COCA) joins COAD and READ in a unique group. This revealed a high-grade of convergence between these two carcinomas [36].

To assess the performances of our methodology, we performed a comparison with Subpathway-GM [24], Subpathway-GMir [27], and DEsubs [26]. Therefore, we ran all competitors, using parameters as suggested from their respective manuscripts, on expression data taken from TCGA, and the results of the enrichment, using the KEGG pathways database, were collected, ranked on the basis of the reported *p*-values, and compared. Since DEsubs returns only the set of significant terms enriching a subpathway, without reporting its *p*-value, we decided to rank each term by the number of subpathways in which such a term is significant.

We opted to run a comparison using only the KEGG pathways database, since the correct identification of the involved pathophysiological process in a subpathway is crucial for subsequent analysis steps. To this end, pathway representing pathological states were removed from enrichment results. All methodologies have been tested using the same set of pathways, enrichment terms, and expression data, although SubPathway-GMir experienced issues when ranking non-metabolic pathways.

The selection of NoIs for SPECifIC was done by using the semi-automated procedure as described in the “Materials and Methods” section. In order to run SPECifIC, our software implementation was used to compute the substructures present in all stages of the aforementioned cancers (BRCA and COAD). Then, for each statistically significant subpathway, functional enrichment was performed, and all pathway terms were collected (regardless of their *p*-value in order to analyze non-significant results). If a term enriched more substructures, the minimum *p*-value among all subpathways was used. The resulting set of terms, ranked by *p*-value, was used, and the top-20 pathways were further analyzed to determine the performances of our methodology (Table 1).

To assess disease-specificity of the results, we computed metrics on disease genes found in the substructures detected by the methodologies, and compared such values with those obtained by applying the same metrics on KEGG pathways. Disease genes were obtained from [37,38] and Genetic Association Database (GAD) [39], removing all genes not present in at least one pathway. The main metrics are: (i) the average distance between a disease gene and a substructure, and (ii) the average distance between pairs of disease genes within the substructures. The first metric is a specificity index of the results, as subpathways closest to disease genes have a greater likelihood of being involved in some way. The second metric is an indication of the characteristic of the genes associated with the same disease to show close trends and common functions, although this is not always an indication of biological relevance. In Table 2, we present the results of the two metrics along with the total number of nodes present in the substructures, and the number of nodes whose expression appears to be significantly differentially expressed (p<0.01). The same table also reports the number of disease genes contained in at least one subpathway and the number of reachable pairs by a directed path. This latter value is critical to properly weigh the second metric. Given that the introduction of miRNAs topologically changes pathways, we also computed the same metrics removing (Subpathway-GM and DESubs do not integrate any information on miRNA-target interactions when computing subpathways.) such elements (Table S1). Our findings show that SPECifIC detects subpathways significantly closer to disease genes with respect to the other approaches (*p*-values computed by Wilcoxon rank-sum test available in Tables S11 and S12). Although it is possible to observe an increase in the average distance between internal pairs of disease genes, these latter values are lower than those observed in KEGG pathways (*p*-values computed by Wilcoxon rank-sum test available in Tables S13 and S14), indicating a potential higher specificity. By removing miRNAs from substructures, we can observe similar results achieved by the compared methodologies. This is an indication of the specificity of the results obtained by the compared methods, although it is not an index of biological reliability.

Finally, due to the high redundancy of biological pathways (different pathways often use the same subpathways in similar roles), we performed an enrichment using disease terms taken from DisGeNET [37,38], in order to evaluate which kind of diseases share the subpathways identified for our two cancer types. The results are available in Table S2.

## 3. Discussion

**Figure 1:**
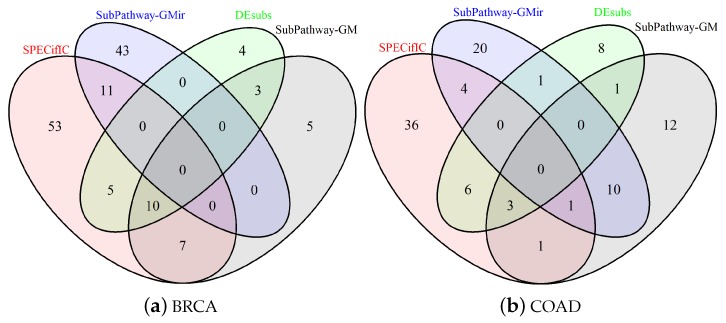
Comparison of the results obtained by SubPathway ExtraCtor and enrICher (SPECifIC), Subpathway- GM [24], Subpathway-GMir [27], and DEsubs [26] for the two datasets: breast invasive carcinoma (BRCA) (**a**) and colon adenocarcinoma (COAD) (**b**). The Venn diagrams have been obtained considering only pathways for which the reported *p*-value was significant (p<0.01).

Disease-specific subpathways extraction is a fundamental process to accurately study processes at the basis of pathological phenotypes. From these substructures, the identification of significantly involved pathways is necessary to determine if the identified changes are at the origin of the disease or a secondary consequence. Furthermore, determining redundant pathways that lead to the same outcome is of great interest especially for precision medicine to identify and, if necessary, prevent drug resistance phenomena. In the following, we will provide a description of the results obtained from SPECifIC in comparisons with the other methods: Subpathway-GM [24], Subpathway-GMir [27], and DEsubs [26]. Complete results are available in Supplementary Materials. We ran all competitors on expression data taken from TCGA and the results of the enrichment were collected using KEGG pathways database. For each method, we selected and considered only the top-20 statistically significant pathway terms (p<0.01), and compared the four methodologies on the basis of such lists (Tables S3–S10). In Figure 1a,b we report Venn diagrams concerning the overlapping of the selected pathways in BRCA and COAD.

### 3.1. Analysis of BRCA Results Using SPECifIC

SPECifIC identified 163 potential pathways associated with BRCA, of which 74 are statistically significant (*p* < 0.01). The list is reported in Table 1 (complete results available in Table S7). The wideness of this subset of pathways is to be considered a reflection of the fact that distinct oncogenic alterations and pathways are associated with diverse breast cancer subtypes [40]. Literature data confirm the involvement of a substantial fraction of this subset of pathways in various BRCA subtypes. First, alterations in gene expression levels within the estrogen and ErbB signaling pathways

play a pivotal role in BRCA development and progression as mentioned above, and dysregulation of various components belonging to these two pathways are themselves the cause of acquired endocrine resistance, typical of many BRCA cases [41]. The involvement of prolactin signaling pathway in breast cancer development is also well documented, and a few works highlight an important cross-talk with the estrogen signaling pathway [42]. Activation of EGFR tyrosine kinase inhibitor resistance characterizes most breast cancer patients also [43], along with the overexpression of CYP19A1, an enzyme with a key role in the steroid hormone biosynthesis, metabolizing *testosterone* into *estradiol* and *androstenedione* to *estrone*, whose dysregulation is relevant to estrogen-dependent pathologies [44]. Focal adhesion [45,46], platelets activation [47], chemokine signaling pathway [48], Phospholipase D signaling pathway [49], and PPAR signaling pathway [50] are overrepresented in this type of cancer as well as in the other types.

Besides the well-known BRCA-associated pathways, scientific evidence exists for several other ones provided by SPECifIC. For example, considering metabolism of xenobiotics and chemical carcinogenesis, CYP1A1 and CYP1B1 genes were found to be highly expressed in a significant number of BRCA individuals [51]. Under physiologic conditions, these genes are expressed at a low level, or are totally suppressed, in a tissue-specific manner [52]. Differently, in breast cancer, CYP1A1 regulates proliferation and survival of tumor cells, while CYP1B1 indirectly causes the generation of free radicals. Regarding the involvement of the insulin signaling pathway in BRCA, literature data indicate that insulin receptors (INSR) are usually overexpressed in BRCA patients—more frequently the INSR isoform A [53]—and a recent work also demonstrated that *flotillin* expression is positively correlated with that of ERBB2 in BRCA. In particular, *flotillin-2* emerged as a potential predictor of prognosis in HER2-amplified breast cancer [54]. A substantial body of evidence proves that neurotrophin signaling pathway is implicated in the stimulation of breast cancer cell growth [55,56]. A significant dysregulation of oxytocin receptors (OXTR) in BRCA subtypes has also been reported [46,57,58].

Finally, regarding the remaining four pathways (drug metabolism–cytochrome P450, drug metabolism–other enzymes, linoleic acid metabolism and phenylalanine metabolism) scarce or no evidence was found in literature.

### 3.2. Comparison for BRCA-Related Pathways

SubPathway-GM was able to identify 25 potential pathway terms, of which 14 were shared with SPECifIC (Table S8). Cell cycle [59], focal adhesion, jak/stat signaling pathway, tight junction [60,61], adherens junction [62], gap junction [63] and regulation of actin cytoskeleton [64], well known cancer-related pathways, were top-ranked by this method. Of these pathway terms, a few were recognized as statistically non-significant by SPECifIC. In addiction, SubPathway-GM returned some potentially BRCA-related pathways. For example, deregulation of embryonic signalling pathways (notch signaling, Hedgehog, Wnt, and transforming growth factor beta signaling pathways) is recognized as having a strong impact in human cancers, including BRCA [65]. Though our method missed this pathway, it identified the Wnt signaling pathway as statistically significant (but not as top ranked), while SubPathway-GM classified it as statistically non-significant. Another result returned by SubPathway-GM is the Mitogen-activated protein kinase (MAPK) signaling pathway. Indeed, even if it is not a directly a BRCA-correlated pathway, recent works proved a strong synergistic effect with notch signaling pathway [66], and PIK3/Akt signaling pathway [67], enhancing the process of transformation and proliferation of breast cancer cells in certain BRCA subtypes.

DEsubs identified 22 terms, of which 13 were in common with our approach (Table S10). Most of these 22 pathways are well known cancer-associated pathways. It is worth noting that DEsubs could classify the Hedgehog signaling pathway as relevant for BRCA together with the Notch signaling pathway. This method also identified the ECM receptor interaction as BRCA-associated [68]. SPECifIC and SubPathway-GM classified it as statistically non-significant. Finally, DEsubs also returned Gonadotropin-releasing hormone (GnRH) and Vascular endothelial growth factor (VEGF) signaling pathways as top ranked in BRCA, and literature confirms these results [69,70]. SPECifIC also classified them as significantly BRCA-associated (though not included in the top 20 list), whereas SubPathway-GM identified them as statistically non-significant.

SubPathway-GMir was able to identify 53 potential pathways, of which only six were shared with our technique (Table S9). This method showed some shortcomings. First, it has to be noticed that it is able to identify just metabolic pathways. Even though several identified pathways are effectively proved to be altered in BRCA [71,72]. It has to be noticed that results obtained by SubPathway-GMir disagreed with results yielded by the other three methods.

### 3.3. Analysis of COAD Results Using SPECifIC

SPECifIC identified 165 potential pathways associated with CRC, of which 31 are statistically significant (*p* < 0.01). The list is reported in Table 1 (complete results available in Supplementary Table S3). Interestingly, half of these top ranked pathways are shared with those found in BRCA. These results are a practical demonstration of the high redundancy of biological pathways. Literature data confirm the reliability of a significant portion of these results. Typical cancer-related pathways, such as Apoptosis pathway [73], MTOR signaling pathway [74], RAS signaling pathway [75], Phospholipase-D signaling pathway [76,77], and HIF-1 signaling pathway [78,79] were returned by SPECifIC. Several works proved that expression of many cytochrome p450 enzymes is altered in CRC [80,81], thus impacting the physiological activity of pathways like metabolism of xenobiotics by cytochrome p450, chemical carcinogenesis and drug metabolism—cytochrome p450. EGFR tyrosine kinase inhibitor resistance in CRC can be developed regardless of the treatment, due to mutations on KRAS, or amplification of MET [82]. Concerning the linoleic acid metabolism, it is proved that linoleic and α-linolenic acid levels are lower in the cell membranes of colorectal cancer cells [83]. For the other pathways, less evidence exists in literature. For example, nothing is known concerning the role of Rap1 signaling pathway in CRC. However, two recent papers showed that miR-100 and miR-139 strongly decreased colorectal carcinoma cell proliferation by directly targeting RAP1B [84,85]. Some data suggest that HSD17B1, an enzyme catalyzing the conversion from *estrone* into *estradiol*, highly expressed in the colonic epithelium, may contribute to sex steroid-mediated effects on CRC development [86]. Two recent works demonstrated that hyper- and hypothyroidism are associated with modestly elevated risk of CRC [87,88]. Furthermore, deregulation of deiodinases (D2–D3) perturb Wnt signaling [89] in CRC patients.

Literature information relative to the involvement in CRC of endocrine resistance, progesterone mediated oocyte maturation, melanogenesis and platinum drug resistance pathways are scarce or absent.

### 3.4. Comparison for COAD-Related Pathways

SubPathway-GM was able to identify 27 potential terms, of which only three were shared with SPECifIC (Table S4). Alterations in PPAR signaling, tight junction, focal adhesion, apoptosis, MAPK signaling and Wnt signaling are typically associated with CRC cells [90,91,92,93,94,95,96]. Moreover, this method classified nine top ranked metabolic pathways as potentially involved in this disease. Recent scientific works based on gene expression analysis and metabolomics reported results showing a wide metabolic reprogramming in colorectal cancer cells. More precisely, levels in enzymes and metabolites involved in arginine and proline metabolism, alanine aspartate and glutamate metabolism, tyrosine metabolism, pyruvate metabolism, fatty acid degradation and butanoate metabolism were confirmed to be significantly altered [45,97,98,99,100,101]. Concerning the natural killer cell mediated cytotoxicity, it has been proved that in a substantial fraction of cases, natural killer (NK) cells belonging to CRC-affected patients show a significant downregulation of several surface receptors [102,103]. No information was found in literature regarding aldosterone regulated sodium reabsorption and oocyte meiosis.

DEsubs identified 19 terms, of which five were in common with our approach (Table S6). A small fraction of these pathways was discussed above for SPECifIC and SubPathway-GM. A recent scientific work showed that expression of FcϵRI α- and γ-chains were significantly upregulated in 70% of colon cancer patients, suggesting that FcϵRI may contribute to the pathophysiology of the gastrointestinal tract [104]. A growing body of evidence showed that inflammatory cells usually produce large amounts of pro-tumorigenic cytokines, such as *TNF*, *Interleukin-6* and *Interleukin-23*, which cause cell proliferation and tumor progression in colorectal cells. In addition, it was proved that NF-κB signaling, activated through TNF-α, strongly induces migration and accumulation of mesenchymal stem cells at CRC-tumor sites [105,106]. No significant information was found about relationships between CRC and B-cell receptor signaling pathway, leukocyte transendothelial migration, long-term depression pathway or GNRH signaling pathway.

SubPathway-GMir was able to identify 35 potential metabolic pathways, of which only three shared with our technique (Table S5). Considering its top 20 pathways, three were identified as statistically significant by SPECifIC, five were classified as statistically significant by SubPathway-GM and only one was shared with DEsubs. Albeit the relation with CRC is validated for a considerable number of these pathways according to the scientific works discussed above for SubPathway-GM, literature information about the remaining fraction of returned metabolic pathways were scarce or not found.

## 4. Materials and Methods

### 4.1. Overview of the Methodology

Our algorithm performs the extraction and enrichment of disease-specific subpathways starting from a set of Nodes of Interest (NoIs). A user may specify his own NoIs or employ an automated selection procedure. The methodology performs computation in four main steps: (i) construction of the meta-pathway; (ii) execution of the MITHrIL algorithm to compute nodes perturbations and *p*-values; (iii) automatic selection of NoIs (if no user-specified ones available), extraction of subpathways, and (iv) enrichment analysis. This is available through a web interface that allows a user to select one disease, identify a set of NoIs, and visualize the results.

#### 4.1.1. Extending MITHrIL

To analyze pathways and determine the impact of differentially expressed genes (miRNAs), we employed the MITHrIL algorithm [21] for its features and performances. The MITHrIL algorithm, in fact, annotates pathways with miRNA-target interactions, therefore obtaining significantly better performances over other competitors, as stated in Alaimo et al. [21].

**Meta-pathway construction.** In order to extract human-specific subpathways, all KEGG pathways were merged to build a single directed network using a two step procedure. First, all disease-specific pathways were removed to form a reference set of pathways. Therefore, all nodes and edges present in the reference set were put together, removing duplicates, building a meta-pathway, which represents the human organism.

Subsequently, the meta-pathway was annotated with experimentally validated miRNA-target interactions using the procedure described in Alaimo et al. [21], and a perturbation analysis is performed by employing differentially expressed genes and miRNAs for each disease.

Such an analysis is computationally intensive. Therefore, to reduce computational resources, all nodes of the meta-pathway were sorted using a DFS-like (Depth-First Search) algorithm [107] to find an approximation of the topological ordering of the nodes of the graph. The perturbation is therefore computed on each node by following such an ordering, caching the results for each call, allowing a reduction of the number of recursive calls, and improving the performances.

**Computing nodes’ *p*-values.** The extraction of subpathway is based on the search of statistically significant substructures within the meta-pathway. This computation is based on the statistical significance of each pathway node, which is the probability of observing a perturbation factor lower than the one computed for the null model.

Therefore, let *n* be a node in the meta-pathway and $PF\left(n\right)$ its perturbation factor. As in Alaimo et al. [21], we randomly assign Log-Fold-Changes to the nodes of the meta-pathway in order to evaluate a set of random perturbations $PF^{R}\left(n\right)=\left\{PF_{1}^{R}\left(n\right),\cdots ,PF_{k}^{R}\left(n\right)\right\}$. The *p*-value for *n* is estimated as:

(1) $$p\left(n\right)=\frac{\left|\left\{x\in PF^{R}\left(n\right)|x\geq PF\left(n\right)\right\}\right|}{k}.$$

*p*-values are therefore adjusted on multiple hypothesis, and the results are stored for subsequent computations.

#### 4.1.2. Subpathway Extraction

The disease-specific subpathway extraction process starts from the previously built meta-pathway annotated with perturbations and *p*-values computed by means of the extended MITHrIL algorithm. Our methodology is, therefore, able to extract five types of substructures starting from a set of NoIs: (i) paths; (ii) tree; (iii) neighborhoods; (iv) induced sub-graphs; and (v) communities of induced sub-graphs. The computation of trees, path and neighborhoods is obtained by running a modified version of the Breadth-first search (BFS) algorithm [107] applied to the meta-pathway. The tree and path topologies obtained using our system are then merged into larger connected components to obtain induced sub-graphs and communities.

The BFS algorithm is a search method in a graph, which, starting from a node (source), obtains all the paths to the other reachable nodes of the graph. The algorithm has been modified to take into account the *p*-value of the nodes: each path from the source is extended with a new node if its *p*-value is greater than a user-specified threshold. Paths identified by running a BFS search make up a tree whose root is the source node.

For each subpathway identified by the algorithm, a *p*-value can be computed by aggregating its nodes *p*-values employing the Empirical Brown’s method [108]. Given a set of *k* dependent *p*-values, Brown [109] estimated that:

(2) $$\psi =-2\sum_{i=1}^{k}ln\left(p_{i}\right)∼c\chi_{2f}^{2},$$

where $p_{i}$ is the *i*-th *p*-value, $f={E\left[\psi \right]}^{2}\;/\;var\left[\psi \right]$, and c=k/f. Therefore, by employing a scaled chi-squared distribution with 2f degrees of freedom, we can compute a combined *p*-value for a *k*-nodes subpathway.

The extraction algorithm also returns subgraphs induced from the BFS visit tree, and the community obtained by merging all induced sub-graphs.

All results are then filtered by removing those subpathways that have a number of nodes below a minimum user-specified threshold, and *p*-values obtained using Empirical Brown’s method are adjusted on multiple hypotheses using the Holm–Bonferroni [110] correction to control a family-wise error rate.

### 4.2. NoIs Selection

The selection of NoIs is a fundamental process for the correct extraction of disease-specific sub-pathways. For this purpose, a semi-automated procedure has been devised for the unbiased selection of NoIs. The user specifies a significance threshold for pathways. Then, all significant pathways are selected using such threshold, and all nodes of these pathways are analyzed. The nodes that exhibit a non-significant *p*-value are discarded. Finally, all duplicates are removed, and a preliminary list of NoIs is generated. The sub-pathway extraction procedure is then applied to such a list, and each resulting path is analyzed to identify overlapping results. The latter are removed in order to keep only unique results, removing duplicates.

#### Subpathway Enrichment Analysis

All extracted subpathways are examined by means of enrichment analysis in various biological and pharmacological features. We are able to estimate associations with (i) pathways; (ii) gene ontology (GO) terms; (iii) diseases; and (iv) drugs. The enrichment analysis is performed by employing the methodology of Li et al. [24]. Given a sub-graph *S* of the meta-pathway, we can compute a *p*-value $p\left(S,t\right)$ for a term *t* as:

(3) pS,t=1−∑i=1kMi·N−Mn−iNn,

where *k* is the number of nodes in *S* annotated with *t*, *M* is the number of nodes in the meta-pathway annotated with *t*, *N* is the number of annotated nodes in the meta-pathway, and *n* is the number of annotated nodes in *S*. Finally, all *p*-values are adjusted on multiple hypotheses using the Benjamini–Hochberg method [111] to control the false discovery rate, and all terms with a *p*-value lower than an user-specified threshold are kept by the analysis.

Since some terms might annotate few nodes with respect to the total, they could appear significant for a subpathway even if they annotate a single node. Therefore, to avoid this shortcoming, we chose to exclude annotations found in less than a third of a subpathway nodes.

### 4.3. Web Interface

In order to ensure broad accessibility and usability, our algorithm has been implemented in a web application available at [29]. The service has been mainly developed using PHP, HTML and JavaScript, with the help of the Laravel framework [112]. To increment performances, the component that deals with substructures computation has been developed in Java, and the functional enrichment procedure has implemented using the *R* statistical environment [113]. The web application also employs Cytoscape.js [114] for displaying subpathways. Furthermore, to minimize user waiting time, all the computations are cached to be reused; therefore no user private data are stored. In Table 3, we report all sources employed for the enrichment analysis.

#### 4.3.1. Expression Data Sources

Our platform has been designed to perform disease-specific analysis on expression data provided by The Cancer Genome Atlas. We collected all patients’ expression profiles of genes (RNASeqV2 obtained through platforms Illumina Genome Analyzer and Illumina HiSeq) and miRNAs (miRNASeq obtained through platforms Illumina Genome Analyzer and Illumina HiSeq) using the TCGA dump provided in Alaimo et al. [21]. Therefore, we removed all tumor samples for which no controls were available, obtaining a dataset of 10 distinct tumor pathologies divided by stage (see Table 4 for more details).

On the aforementioned dataset, we applied the pipeline described in Alaimo et al. [21] in order to pre-compute differentially expressed genes and meta-pathway perturbations using the extended version of the MITHrIL algorithm.

#### 4.3.2. Analysis Workflow

**Figure 2:**
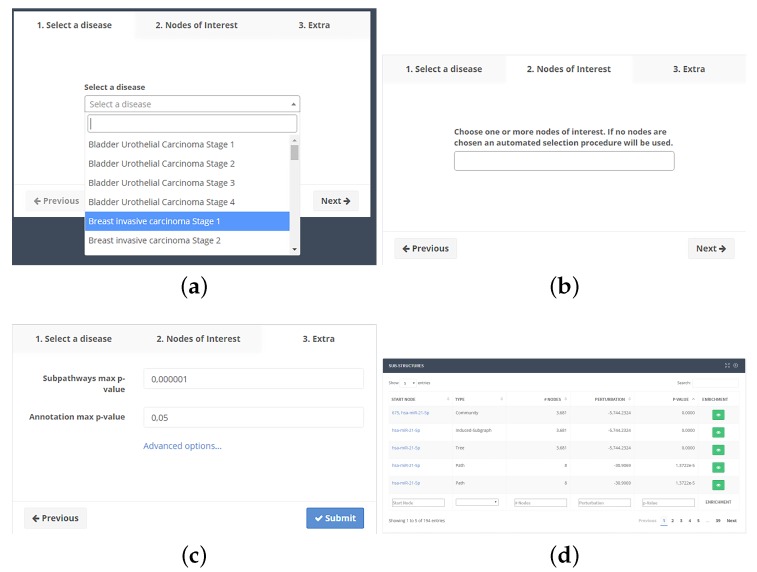
Subpathway extraction analysis workflow. (**a**) After selecting a disease and a stage among those available into the drop-down box, (**b**) the user can optionally choose one or more Nodes of Interest (NoIs); (**c**) and, after modifying the optional parameters, the job can be submitted to our servers. After processing, (**d**) a table will be shown with a list of found substructures, giving the ability to perform the functional annotation.

To use the web application, the user selects one of the available pathologies (Figure 2a). After confirming the selection, one or more nodes of interest can be optionally selected (Figure 2b), paying attention to the *p*-value of each NoI. If the *p*-value for a node is greater than the maximum threshold, it will be discarded from the analysis. The user can also vary *p*-value thresholds and the minimum number of nodes in substructures (Figure 2c). From the same screen, the user can also select whether the search should proceed backward from each NoI or forward.

Clicking on the submission button, the job will be put into a computing queue. At the end of computation, a table (Figure 2d) with the list of substructures will be shown. Such table will show the type of structure, the source node, the number of nodes, the *p*-value adjusted by the Holm–Bonferroni method and a button, which displays the functional enrichment results. The user will be able to download all results as a tab-separated text file.

**Figure 3:**
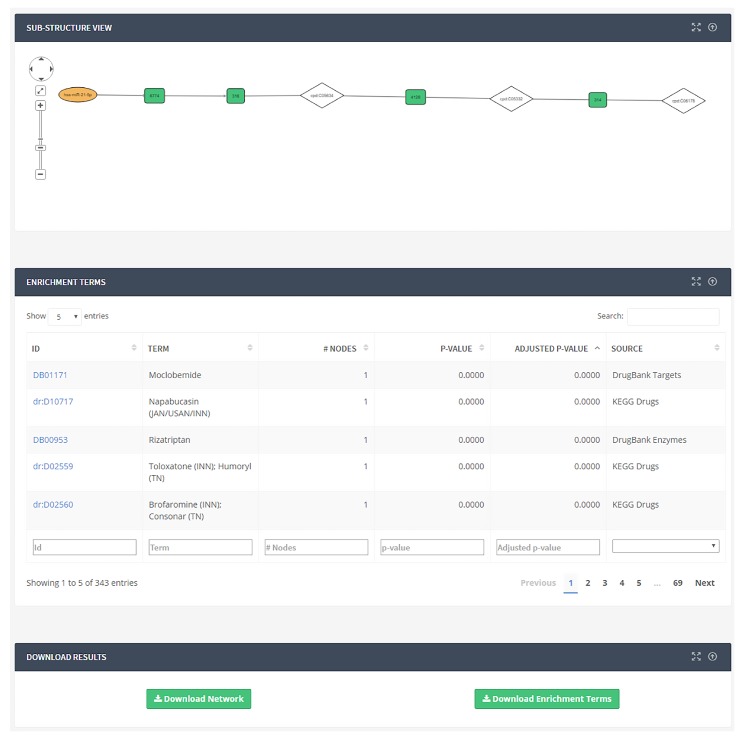
Results of the functional annotation analysis of a substructure. The results are shown in a table together with the graph representing the structure itself. In the table, we show the term identifier in the source database, the term description, the number of annotated nodes, the *p*-value, the adjusted *p*-value, and the source of the term. By clicking on any term identifier, the annotated nodes will be highlighted in the graph. The user is also able to download annotation results in a tab-separated file and the network in XGMML format.

Functional enrichment results are displayed in a page together (Figure 3) with a Cytoscape.js-based viewer to see substructures. The list of terms is sorted by adjusted *p*-value. Two buttons will allow the download of the enrichment (tab-separated text file) and the substructure (XGMML format). The list of terms is displayed in a table together with their identifier, number of annotated nodes, *p*-value, adjusted *p*-value by applying the Benjamini–Hochberg method [111], and the source database.

All tables in the web interface give the user the ability to filter results in order to facilitate subsequent analysis.

## 5. Conclusions

In this paper, we proposed a novel methodology, SPECifIC (SubPathway ExtraCtor and enrICher), for extracting, visualizing, and enriching substructures obtained from a meta-pathway built by combining KEGG pathways. The methodology extends MITHrIL [21] to allow efficient nodes’ perturbation and statistical significance computation, and leverages on the increased precision given by the addition of post-trascriptional regulatory elements, such as microRNAs. Starting from expression data of pathological samples, the method is able to extract statistically significant disease-specific subpathways, and perform functional enrichment, allowing further evaluations.

The technique has been compared with three other methodologies, Subpathway-GM [24], Subpathway-GMir [27], and DEsubs [26], on two expression datasets (BRCA and COAD) taken from The Cancer Genome Atlas.

Our findings show that SPECifIC is able to produce results in agreement with the current knowledge of the pathophysiological processes of BRCA and COAD, and with the results produced by the three competitors. In addition, the pathways, which the method discards, do not show any significant correlation with the two pathological phenotypes, according to literature. However, our results are larger than other competitors, leading to the need of a better filters in order to benefit more from them.

On the other hand, our analysis clearly highlights that, although all the compared methods give in general biologically sound results, their consensus is certainly small.

Future developments should also integrate information coming from expressions of other classes of non-coding RNAs, whose alteration has been correlated in different pathological phenotypes, and the introduction of gene mutations and copy number variation in order to detect more disease-specific subpathways based on the pattern of altered genes rather than their expression.

## Figures and Tables

**Table 1:** Top-20 pathways obtained for breast invasive carcinoma (BRCA) and colon adenocarcinoma (COAD) after subpathways extraction and enrichment performed by means of SubPathway ExtraCtor and enrICher (SPECifIC). All terms were first ranked by adjusted *p*-value and the top-20 significant results were taken for further analysis.

BRCA		COAD	
Pathway	*p*	Pathway	*p*
metabolism of xenobiotics by cytochrome p450	0	metabolism of xenobiotics by cytochrome p450	0
steroid hormone biosynthesis	0	drug metabolism cytochrome p450	0
drug metabolism cytochrome p450	0	chemical carcinogenesis	0
chemical carcinogenesis	0	steroid hormone biosynthesis	0
drug metabolism other enzymes	0	drug metabolism other enzymes	0
linoleic acid metabolism	0	linoleic acid metabolism	0
longevity regulating pathway	$3.27\times 10^{-8}$	ppar signaling pathway	0
egfr tyrosine kinase inhibitor resistance	$2.17\times 10^{-7}$	phenylalanine metabolism	0
endocrine resistance	$2.35\times 10^{-7}$	estrogen signaling pathway	$1.09\times 10^{-30}$
rap1 signaling pathway	$5.32\times 10^{-7}$	chemokine signaling pathway	$1.28\times 10^{-30}$
progesterone mediated oocyte maturation	$5.39\times 10^{-7}$	erbb signaling pathway	$8.64\times 10^{-29}$
hif 1 signaling pathway	$5.89\times 10^{-7}$	phospholipase d signaling pathway	$1.11\times 10^{-27}$
melanogenesis	$6.15\times 10^{-7}$	neurotrophin signaling pathway	$4.63\times 10^{-27}$
apoptosis	$1.27\times 10^{-6}$	insulin signaling pathway	$7.95\times 10^{-26}$
platinum drug resistance	$1.27\times 10^{-6}$	egfr tyrosine kinase inhibitor resistance	$2.76\times 10^{-25}$
phospholipase d signaling pathway	$1.30\times 10^{-6}$	prolactin signaling pathway	$1.20\times 10^{-24}$
mtor signaling pathway	$1.55\times 10^{-6}$	oxytocin signaling pathway	$5.67\times 10^{-24}$
ras signaling pathway	$1.55\times 10^{-6}$	platelet activation	$5.67\times 10^{-24}$
thyroid hormone signaling pathway	$3.13\times 10^{-6}$	endocrine resistance	$5.87\times 10^{-24}$
erbb signaling pathway	$3.32\times 10^{-6}$	focal adhesion	$6.00\times 10^{-24}$

**Table 2:** Metrics computed for the subpathways disease-specificity assessment of the two datasets in our case study. The table shows the number of substructures nodes, the number of significant nodes (p<0.01), the number of disease genes, the number of significant disease genes (p<0.01), the number of reachable pairs of disease genes within subpathways, the average distance between a disease gene and a substructure ${}^{†}$, and the average distance between disease genes contained within each substructure ${}^{‡}$. The results are compared with a reference computed directly in Kyoto Encyclopedia of Genes and Genomes (KEGG) pathways.

		# Nodes		# Disease Genes				
**Dataset**	**Algorithm**	p<0.01	**All**	p<0.01	**All**	**Reachable Pairs**	†	‡
	*KEGG Pathways*	1009	7121	30	104	283	-	7
	*SPECifIC*	466	466	15	15	6	**1.78**	3
**BRCA**	*SubPathway-GM*	101	214	9	14	6	1.89	3
	*SubPathway-Gmir*	142	722	4	8	1	2.09	2
	*DESubs*	34	34	0	0	0	2.48	-
	*KEGG Pathways*	1009	7121	11	81	490	-	9
	*SPECifIC*	486	486	9	9	6	**1.67**	4
**COAD**	*SubPathway-GM*	59	173	3	8	4	2.04	3
	*SubPathway-Gmir*	158	248	4	7	9	2.20	2
	*DESubs*	6	6	0	0	0	2.97	-

**Table 3:** List of terms sources used for the enrichment phase. All sources are grouped by category. For each source, we report the name, the number of terms, and the number of enriched nodes.

Category	Source	# Terms	# Nodes
**Diseases**			
	DisGeNET [37,38]	7607	2978
	GAD [39]	403	1519
	KEGG [2,3,4] Diseases	1278	1234
	OMIM [115]	89	518
**Drugs**			
	Drugbank [116] Carriers	247	7
	Drugbank [116] Enzymes	797	180
	Drugbank [116] Targets	4815	1494
	Drugbank [116] Transporters	560	18
	KEGG [2,3,4] Drugs	3793	706
**Gene Ontology**			
	GO [117,118] Biological Processes	11,386	4850
	GO [117,118] Cellular Component	1545	4852
	GO [117,118] Molecular Function	4146	4832
**Pathways**			
	KEGG [2,3,4] Pathways	310	4904

**Table 4:** List of cancer types extracted from TCGA with their codes, number of case and control samples, and subcategories

Code	Cancer Type	Control Samples	Case Samples	Case Samples Categories
BLCA	Bladder Urothelial Carcinoma	19	193	Stage I, II, III, IV
BRCA	Breast invasive carcinoma	86	642	Stage I, II, III, IV, X
COAD	Colon adenocarcinoma	8	389	Stage I, II, III, IV
KICH	Kidney Chromophobe	25	66	Stage I, II, III, IV
KIRC	Kidney renal clear cell carcinoma	71	224	Stage I, II, III, IV
LUAD	Lung adenocarcinoma	19	388	Stage I, II, III, IV
LUSC	Lung squamous cell carcinoma	37	247	Stage I, II, III, IV
PRAD	Prostate adenocarcinoma	50	191	Category 6, 7, 8, 9, 10
READ	Rectum adenocarcinoma	3	150	Stage I, II, III, IV
UCEC	Uterine Corpus Endometrial Carcinoma	14	231	Stage I, II, III, IV
	All Samples	332	2721	

## Supplementary Materials

The following are available online at www.mdpi.com/2311-553X/3/2/20/s1, Tables S1–S14 Detailed comparison of the predictions made by the four algorithms on the COAD and BRCA datasets.

## Abbreviations

The following abbreviations are used in this manuscript:

TCGA	The Cancer Genome Atlas
CRC	Colorectal Carcinoma
BRCA	Breast Invasive Carcinoma
COAD	Colon Adenocarcinoma
READ	Rectal Adenocarcinoma
PF	Perturbation Factor
MITHrIL	Mirna enrIched paTHway Impact anaLysis
SPECifIC	SubPathway ExtraCtor and enrICher
SPIA	Signaling Pathway Impact Analysis
TEAK	Topology Enrichment Analysis frameworK
PARADIGM	PAthway Representation and Analysis by Direct Reference on Graphical Models
DEG	Differentially Expressed Gene
IF	Impact Factor
