# Supplementary material for: Detecting Disease Specific Pathway Substructures through an Integrated Systems Biology Approach
Source: Noncoding RNA. 2017 Apr 19;3(2):20. doi: 10.3390/ncrna3020020 (PMC5831934; doi:10.3390/ncrna3020020)
Supplement: Supplementary file 1 [file ncrna-03-00020-s001.pdf]

Supplementary Materials: Detecting disease specific pathway substructures through an integrated systems biology approach

Salvatore Alaimo [1], Gioacchino Paolo Marceca [1], Alfredo Ferro [1] and Alfredo Pulvirenti[1]*

1. Supplementary Tables

Table S1. Metrics computed for the subpathways disease-specificity assessment of the two datasets in our case study when microRNAs are removed. The table shows the number of substructures nodes, the number of significant nodes ($p < 0.01$), the number of disease genes, the number of significant disease genes ($p < 0.01$), the number of reachable pairs of disease genes within subpathways, the average distance between a disease gene and a substructure†, and the average distance between disease genes contained within each substructure‡. The results are compared with a reference computed directly on KEGG pathways.

		# Nodes		# Disease Genes				
**Dataset**	**Algorithm**	$p < 0.01$	**All**	$p < 0.01$	**All**	**Reachable Pairs**	†	‡
**BRCA**	*KEGG Pathways*	983	6688	30	104	156	-	3
	SPECifIC	247	247	15	15	2	1,83	2
	SubPathway-GM	101	214	9	14	6	2,64	3
	SubPathway-Gmir	135	682	4	8	1	2,76	2
	DESubs	34	34	0	0	0	1,71	-
**COAD**	*KEGG Pathways*	995	6688	11	81	88	-	3
	SPECifIC	139	139	9	9	2	1,97	2
	SubPathway-GM	59	173	3	8	4	2,19	3
	SubPathway-Gmir	131	221	4	7	9	2,96	2
	DESubs	6	6	0	0	0	2,4	-

Table S2. Enrichment computed on the subpathways extracted by SPECifIC on the two cancer types (BRCA and COAD) using terms coming from the DisGeNET [38,39] database. Only the significant terms ($p < 0.01$) were kept.

COAD		BRCA	
**Disease**	**Adjusted p-value**	**Disease**	**Adjusted p-value**
Prostatic Neoplasms	0	*Prostatic Neoplasms*	0
Mammary Neoplasms	0	*Mammary Neoplasms*	0
Osteosarcoma	0	*Osteosarcoma*	0
Hepatitis C	0	*Hepatitis C*	0
Torsades de Pointes	0	*Torsades de Pointes*	0
Esophageal Neoplasms	0.0001	*MICROPHTHALMIA. ISOLATED 8*	0
Adenocarcinoma	0.0002	*Prostatic Neoplasms. Castration-Resistant*	0
Colorectal Neoplasms	0.0005	*Neoplasms. Hormone-Dependent*	1.41×10^{-08}
Hypertensive disease	0.0005	*Disorders of Sex Development*	7.05×10^{-08}
Lung Neoplasms	0.0005	*Obesity*	3.38×10^{-06}
Liver carcinoma	0.0011	*Substance-Related Disorders*	0.0002
Reperfusion Injury	0.0012	*Polycystic Ovary Syndrome*	0.0002
		Hypertensive disease	0.0004
		Myocardial Infarction	0.0006
		Stomach Neoplasms	0.0010
		Diabetes Mellitus. Experimental	0.0011
		Colorectal Neoplasms	0.0022

Table S3. Results obtained by employing SPECifIC to extract and enrich COAD subpathways using KEGG pathway terms. The terms are sorted by means of p-value. We also reports if the other methods detect the same term as statistically significant ($p < 0.01$). In italics we report the top-20 pathways used for the comparisons.

**Pathway**	**SPECifIC**	**SubPathway-GM**	**SubPathway-GMir**	**DEsubs**
metabolism of xenobiotics by cytochrome p450	*0*		*Yes*	
steroid hormone biosynthesis	*0*		*Yes*	
drug metabolism cytochrome p450	*0*			
chemical carcinogenesis	*0*			
drug metabolism other enzymes	*0*			
linoleic acid metabolism	*0*			
longevity regulating pathway	3.27×10^{-08}			
egfr tyrosine kinase inhibitor resistance	2.17×10^{-07}			
endocrine resistance	2.35×10^{-07}			
rap1 signaling pathway	5.32×10^{-07}			
progesterone mediated oocyte maturation	5.39×10^{-07}			
hif 1 signaling pathway	5.89×10^{-07}			
melanogenesis	6.15×10^{-07}			Yes
apoptosis	1.27×10^{-06}	Yes		Yes
platinum drug resistance	1.27×10^{-06}			
phospholipase d signaling pathway	1.30×10^{-06}			
mtor signaling pathway	1.55×10^{-06}			
ras signaling pathway	1.55×10^{-06}			
thyroid hormone signaling pathway	3.13×10^{-06}			
erbb signaling pathway	3.32×10^{-06}			Yes
estrogen signaling pathway	3.97×10^{-06}			
inflammatory mediator regulation of trp channels	4.85×10^{-06}			
proteoglycans in cancer	5.00×10^{-06}			
pathways in cancer	7.08×10^{-06}			
platelet activation	9.18×10^{-06}			
chemokine signaling pathway	1.42×10^{-05}	Yes		
gnrh signaling pathway	1.50×10^{-05}			Yes
oxytocin signaling pathway	1.61×10^{-05}			
arachidonic acid metabolism	2.34×10^{-05}		Yes	
pi3k akt signaling pathway	0.0003			
mapk signaling pathway	0.0017	Yes		Yes
insulin secretion	0.5000			
ovarian steroidogenesis	0.5000			
beta alanine metabolism	0.5000			
long term depression	0.5000			Yes
thyroid hormone synthesis	0.5000			
aldosterone synthesis and secretion	0.5000			
gap junction	0.5000			
vegf signaling pathway	0.5000			Yes
b cell receptor signaling pathway	0.5000			Yes
camp signaling pathway	0.5000			
glucagon signaling pathway	0.5000			
micrornas in cancer	0.5000			
regulation of lipolysis in adipocytes	0.5000			
dopaminergic synapse	0.5000			
natural killer cell mediated cytotoxicity	0.5000	Yes		Yes
adrenergic signaling in cardiomyocytes	0.5001			
cgmp pkg signaling pathway	0.5001			
purine metabolism	0.5005		Yes	
fc epsilon ri signaling pathway	1.0000			Yes
cytokine cytokine receptor interaction	1.0000			Yes
inositol phosphate metabolism	1.0000		Yes	Yes
phosphatidylinositol signaling system	1.0000			Yes
leukocyte transendothelial migration	1.0000			Yes
focal adhesion	1.0000	Yes		Yes
toll like receptor signaling pathway	1.0000			Yes
jak stat signaling pathway	1.0000			Yes
regulation of actin cytoskeleton	1.0000			Yes
adipocytokine signaling pathway	1.0000			Yes
starch and sucrose metabolism	1.0000	Yes	Yes	
fatty acid degradation	1.0000	Yes	Yes	
pyrimidine metabolism	1.0000		Yes	
pentose and glucuronate interconversions	1.0000	Yes	Yes	
porphyrin and chlorophyll metabolism	1.0000		Yes	

ascorbate and aldarate metabolism	1.0000		Yes
glutathione metabolism	1.0000	Yes	Yes
galactose metabolism	1.0000		Yes
glycerolipid metabolism	1.0000		Yes
tyrosine metabolism	1.0000	Yes	Yes
glycerophospholipid metabolism	1.0000		Yes
amino sugar and nucleotide sugar metabolism	1.0000	Yes	Yes
fructose and mannose metabolism	1.0000		Yes
citrate cycle tca cycle	1.0000		Yes
pentose phosphate pathway	1.0000		Yes
cysteine and methionine metabolism	1.0000		Yes
ether lipid metabolism	1.0000		Yes
phenylalanine metabolism	1.0000		Yes
arginine and proline metabolism	1.0000	Yes	Yes
pyruvate metabolism	1.0000	Yes	Yes
glycolysis gluconeogenesis	1.0000		Yes
ppar signaling pathway	1.0000	Yes	
tight junction	1.0000	Yes	
calcium signaling pathway	1.0000	Yes	
aldosterone regulated sodium reabsorption	1.0000	Yes	
wnt signaling pathway	1.0000	Yes	
alanine aspartate and glutamate metabolism	1.0000	Yes	
butanoate metabolism	1.0000	Yes	
oocyte meiosis	1.0000	Yes	
cell adhesion molecules cams	1.0000	Yes	
cell cycle	1.0000	Yes	
tgf beta signaling pathway	1.0000	Yes	
fc gamma r mediated phagocytosis	1.0000		
lysine degradation	1.0000		
olfactory transduction	1.0000		
long term potentiation	1.0000		
neurotrophin signaling pathway	1.0000		
nod like receptor signaling pathway	1.0000		
vascular smooth muscle contraction	1.0000		
phenylalanine tyrosine and tryptophan biosynthesis	1.0000		
axon guidance	1.0000		
salivary secretion	1.0000		
insulin signaling pathway	1.0000		
central carbon metabolism in cancer	1.0000		
ampk signaling pathway	1.0000		
sphingolipid signaling pathway	1.0000		
carbohydrate digestion and absorption	1.0000		
viral carcinogenesis	1.0000		
foxo signaling pathway	1.0000		
choline metabolism in cancer	1.0000		
p53 signaling pathway	1.0000		
mrna surveillance pathway	1.0000		
nf kappa b signaling pathway	1.0000		
protein processing in endoplasmic reticulum	1.0000		
apoptosis multiple species	1.0000		
hedgehog signaling pathway	1.0000		
transcriptional misregulation in cancer	1.0000		
circadian entrainment	1.0000		
retrograde endocannabinoid signaling	1.0000		
glutamatergic synapse	1.0000		
gabaergic synapse	1.0000		
renin secretion	1.0000		
gastric acid secretion	1.0000		
pancreatic secretion	1.0000		
abc transporters	1.0000		
dorso ventral axis formation	1.0000		
signaling pathways regulating pluripotency of stem cells	1.0000		
t cell receptor signaling pathway	1.0000		
prolactin signaling pathway	1.0000		
osteoclast differentiation	1.0000		
tnf signaling pathway	1.0000		
th17 cell differentiation	1.0000		
endocrine and other factor regulated calcium reabsorption	1.0000		
vasopressin regulated water reabsorption	1.0000		
autophagy	1.0000		
endocytosis	1.0000		
adherens junction	1.0000		
fatty acid metabolism	1.0000		
proximal tubule bicarbonate reclamation	1.0000		
neomycin kanamycin and gentamicin biosynthesis	1.0000		

carbon metabolism	1.0000
homologous recombination	1.0000
hippo signaling pathway	1.0000
cardiac muscle contraction	1.0000
other types of o glycan biosynthesis	1.0000
ecm receptor interaction	1.0000
protein digestion and absorption	1.0000
ubiquitin mediated proteolysis	1.0000
rna polymerase	1.0000
cytosolic dna sensing pathway	1.0000
neuroactive ligand receptor interaction	1.0000
taste transduction	1.0000
th1 and th2 cell differentiation	1.0000
selenocompound metabolism	1.0000
mineral absorption	1.0000
biosynthesis of amino acids	1.0000
folate biosynthesis	1.0000
intestinal immune network for iga production	1.0000
antifolate resistance	1.0000
hematopoietic cell lineage	1.0000
phototransduction	1.0000
alpha linolenic acid metabolism	1.0000
primary bile acid biosynthesis	1.0000
nucleotide excision repair	1.0000
taurine and hypotaurine metabolism	1.0000
histidine metabolism	1.0000

Table S4. Results obtained by employing SubPathway-GM [1] to extract and enrich COAD subpathways using KEGG pathway terms. The terms are sorted by means of p-value. We also reports if the other methods detect the same term as statistically significant ($p < 0.01$). In italics we report the top-20 pathways used for the comparisons.

Pathway	SPECifIC	SubPathway-GM	SubPathway-GMir	DEsubs
ppar signaling pathway		1.45×10^{-08}		
starch and sucrose metabolism		4.93×10^{-07}	*Yes*	
natural killer cell mediated cytotoxicity		3.86×10^{-06}		*Yes*
pentose and glucuronate interconversions		3.86×10^{-06}	*Yes*	
tight junction		4.53×10^{-06}		
complement and coagulation cascades		*0.0003*		
arginine and proline metabolism		*0.0004*	*Yes*	
apoptosis	*Yes*	*0.0005*		*Yes*
calcium signaling pathway		*0.0006*		
aldosterone regulated sodium reabsorption		*0.0006*		
mapk signaling pathway	*Yes*	*0.0012*		*Yes*
fatty acid degradation		*0.0012*	*Yes*	
focal adhesion		*0.0013*		*Yes*
wnt signaling pathway		*0.0013*		
alanine aspartate and glutamate metabolism		*0.0014*		
butanoate metabolism		*0.0014*		
pyruvate metabolism		*0.0020*	*Yes*	
oocyte meiosis		*0.0026*		
glycosphingolipid biosynthesis lacto and neolacto series		*0.0026*	*Yes*	
tyrosine metabolism		*0.0029*	*Yes*	
chemokine signaling pathway	Yes	0.0030		
glutathione metabolism		0.0044	Yes	
cell adhesion molecules cams		0.0049		
cell cycle		0.0050		
amino sugar and nucleotide sugar metabolism		0.0065	Yes	
tgf beta signaling pathway		0.0065		
one carbon pool by folate		0.0089	Yes	
phosphatidylinositol signaling system		0.0101		Yes
galactose metabolism		0.0131	Yes	
toll like receptor signaling pathway		0.0147		Yes
phenylalanine metabolism		0.0147	Yes	
inositol phosphate metabolism		0.0148	Yes	Yes
fc gamma r mediated phagocytosis		0.0176		
lysine degradation		0.0176		
glycosphingolipid biosynthesis globo and isoglobo series		0.0195		
vegf signaling pathway		0.0218		Yes
fructose and mannose metabolism		0.0230	Yes	

valine leucine and isoleucine degradation		0.0230	Yes	
adipocytokine signaling pathway		0.0253		Yes
glycerophospholipid metabolism		0.0253	Yes	
olfactory transduction		0.0253		
jak stat signaling pathway		0.0282		Yes
citrate cycle tca cycle		0.0282	Yes	
pentose phosphate pathway		0.0282	Yes	
sulfur metabolism		0.0282	Yes	
long term potentiation		0.0286		
glycosphingolipid biosynthesis ganglio series		0.0294	Yes	
progesterone mediated oocyte maturation	Yes	0.0338		
pathways in cancer	Yes	0.0358		
porphyrin and chlorophyll metabolism		0.0437	Yes	
neurotrophin signaling pathway		0.0437		
arachidonic acid metabolism	Yes	0.0467	Yes	
glycerolipid metabolism		0.0467	Yes	
cysteine and methionine metabolism		0.0471	Yes	
drug metabolism cytochrome p450	Yes	0.0482		
long term depression		0.0546		Yes
propanoate metabolism		0.0581		
mtor signaling pathway	Yes	0.0678		
glycolysis gluconeogenesis		0.0700	Yes	
nod like receptor signaling pathway		0.0700		
vascular smooth muscle contraction		0.0700		
phenylalanine tyrosine and tryptophan biosynthesis		0.0700		
glycine serine and threonine metabolism		0.0700	Yes	
tryptophan metabolism		0.0700		
regulation of actin cytoskeleton		0.0812		Yes
purine metabolism		0.0831	Yes	
sphingolipid metabolism		0.0831	Yes	
fatty acid elongation		0.0831		
synthesis and degradation of ketone bodies		0.0831		
circadian rhythm		0.0831		
melanogenesis	Yes	0.0982		Yes
axon guidance		0.0982		
gnrh signaling pathway	Yes	0.1498		Yes
gap junction		0.1558		
pyrimidine metabolism		0.1713	Yes	
steroid hormone biosynthesis	Yes	0.1858	Yes	
salivary secretion		0.1923		
phagosome		0.2123		
ether lipid metabolism		0.2859	Yes	
fc epsilon ri signaling pathway		0.2921		Yes
insulin signaling pathway		0.3107		
mucin type o glycan biosynthesis		0.3288	Yes	
nicotinate and nicotinamide metabolism		0.4014		

Table S5. Results obtained by employing SubPathway-GMir [2] to extract and enrich COAD subpathways using KEGG pathway terms. The terms are sorted by means of p-value. We also reports if the other methods detect the same term as statistically significant ($p < 0.01$). In italics we report the top-20 pathways used for the comparisons.

Pathway	SPECifIC	SubPathway-GM	SubPathway-GMir	DEsubs
metabolism of xenobiotics by cytochrome p450	*Yes*		*0*	
purine metabolism			*0*	
starch and sucrose metabolism		*Yes*	*0*	
fatty acid degradation		*Yes*	*0*	
steroid hormone biosynthesis	*Yes*		1.09×10^{-14}	
pyrimidine metabolism			2.40×10^{-14}	
pentose and glucuronate interconversions		*Yes*	2.68×10^{-14}	
inositol phosphate metabolism			2.90×10^{-14}	*Yes*
porphyrin and chlorophyll metabolism			2.48×10^{-13}	
ascorbate and aldarate metabolism			3.93×10^{-13}	
glutathione metabolism		*Yes*	8.26×10^{-11}	
galactose metabolism			8.26×10^{-11}	
arachidonic acid metabolism	*Yes*		1.18×10^{-09}	
aminoacyl trna biosynthesis			2.28×10^{-09}	
glycerolipid metabolism			2.98×10^{-09}	
valine leucine and isoleucine degradation			3.01×10^{-09}	

tyrosine metabolism	*Yes*	1.37×10^{-08}
glycerophospholipid metabolism		3.75×10^{-08}
amino sugar and nucleotide sugar metabolism	*Yes*	1.71×10^{-07}
fructose and mannose metabolism		7.94×10^{-07}
citrate cycle tca cycle		9.41×10^{-07}
one carbon pool by folate	Yes	2.09×10^{-06}
pentose phosphate pathway		2.89×10^{-06}
cysteine and methionine metabolism		2.89×10^{-06}
glycosphingolipid biosynthesis lacto and neolacto series	Yes	2.89×10^{-06}
ether lipid metabolism		4.29×10^{-06}
phenylalanine metabolism		8.71×10^{-06}
mucin type o glycan biosynthesis		2.15×10^{-05}
sulfur metabolism		4.04×10^{-05}
arginine and proline metabolism	Yes	0.0001
pyruvate metabolism	Yes	0.0001
glycolysis gluconeogenesis		0.0001
sphingolipid metabolism		0.0002
glycosphingolipid biosynthesis ganglio series		0.0013
glycine serine and threonine metabolism		0.0013

Table S6. Results obtained by employing DEsubs [3] to extract and enrich COAD subpathways using KEGG pathway terms. The terms are sorted by means of p-value. We also reports if the other methods detect the same term as statistically significant ($p < 0.01$). In italics we report the top-20 pathways used for the comparisons.

Pathway	SPECifIC	SubPathway-GM	SubPathway-GMir	DEsubs
fc epsilon ri signaling pathway				*67*
cytokine cytokine receptor interaction				*58*
erbb signaling pathway	*Yes*			*46*
b cell receptor signaling pathway				*29*
natural killer cell mediated cytotoxicity		*Yes*		*29*
inositol phosphate metabolism			*Yes*	*29*
phosphatidylinositol signaling system				*29*
leukocyte transendothelial migration				*29*
gnrh signaling pathway	*Yes*			*28*
mapk signaling pathway	*Yes*	*Yes*		*16*
focal adhesion		*Yes*		*16*
long term depression				*12*
toll like receptor signaling pathway				*10*
jak stat signaling pathway				*7*
apoptosis	*Yes*	*Yes*		*6*
vegf signaling pathway				*6*
regulation of actin cytoskeleton				*6*
adipocytokine signaling pathway				*2*
melanogenesis	*Yes*			*1*

Table S7. Results obtained by employing SPECifIC to extract and enrich BRCA subpathways using KEGG pathway terms. The terms are sorted by means of p-value. We also reports if the other methods detect the same term as statistically significant ($p < 0.01$). In italics we report the top-20 pathways used for the comparisons.

Pathway	SPECifIC	SubPathway-GM	SubPathway-GMir	DEsubs
metabolism of xenobiotics by cytochrome p450	*0*		*Yes*	
drug metabolism cytochrome p450	*0*		*Yes*	

chemical carcinogenesis	*0*			
steroid hormone biosynthesis	*0*		*Yes*	
drug metabolism other enzymes	*0*		*Yes*	
linoleic acid metabolism	*0*		*Yes*	
ppar signaling pathway	*0*	*Yes*		*Yes*
phenylalanine metabolism	*0*		*Yes*	
estrogen signaling pathway	1.09×10^{-30}			
chemokine signaling pathway	1.28×10^{-30}	*Yes*		
erbb signaling pathway	8.64×10^{-29}			
phospholipase d signaling pathway	1.11×10^{-27}			
neurotrophin signaling pathway	4.63×10^{-27}	*Yes*		
insulin signaling pathway	7.95×10^{-26}	*Yes*		
egfr tyrosine kinase inhibitor resistance	2.76×10^{-25}			
prolactin signaling pathway	1.20×10^{-24}			
oxytocin signaling pathway	5.67×10^{-24}			
platelet activation	5.67×10^{-24}			
endocrine resistance	5.87×10^{-24}			
focal adhesion	6.00×10^{-24}	*Yes*		*Yes*
inflammatory mediator regulation of trp channels	1.98×10^{-23}			
cholinergic synapse	5.56×10^{-21}			
adrenergic signaling in cardiomyocytes	1.53×10^{-20}			
rap1 signaling pathway	2.43×10^{-20}			
vegf signaling pathway	2.53×10^{-20}			Yes
sphingolipid signaling pathway	2.68×10^{-20}			
natural killer cell mediated cytotoxicity	3.34×10^{-20}			Yes
thyroid hormone signaling pathway	9.45×10^{-19}			
ras signaling pathway	2.83×10^{-18}			
pathways in cancer	5.11×10^{-18}			
fc epsilon ri signaling pathway	1.36×10^{-17}			Yes
b cell receptor signaling pathway	7.78×10^{-17}	Yes		
cgmp pkg signaling pathway	2.27×10^{-16}			
phosphatidylinositol signaling system	7.35×10^{-16}			
choline metabolism in cancer	1.56×10^{-15}			
mtor signaling pathway	2.78×10^{-15}			
foxo signaling pathway	3.66×10^{-15}			
regulation of actin cytoskeleton	4.17×10^{-15}	Yes		Yes
t cell receptor signaling pathway	6.31×10^{-15}	Yes		
aldosterone regulated sodium reabsorption	1.20×10^{-14}			
central carbon metabolism in cancer	2.24×10^{-14}			
longevity regulating pathway multiple species	3.08×10^{-14}			
carbohydrate digestion and absorption	3.96×10^{-14}			
gnrh signaling pathway	4.00×10^{-14}			Yes
proteoglycans in cancer	1.87×10^{-13}			
serotonergic synapse	3.38×10^{-13}			
regulation of lipolysis in adipocytes	4.07×10^{-13}			
leukocyte transendothelial migration	4.29×10^{-13}			
fc gamma r mediated phagocytosis	8.62×10^{-13}	Yes		
signaling pathways regulating pluripotency of stem cells	8.89×10^{-13}			
micrornas in cancer	1.29×10^{-12}			
longevity regulating pathway	1.56×10^{-12}			
progesterone mediated oocyte maturation	1.86×10^{-12}			
long term depression	2.97×10^{-12}			Yes
camp signaling pathway	3.76×10^{-12}			
axon guidance	6.84×10^{-12}			
inositol phosphate metabolism	1.03×10^{-11}		Yes	
viral carcinogenesis	2.21×10^{-11}			
pi3k akt signaling pathway	2.70×10^{-11}			
jak stat signaling pathway	3.09×10^{-11}	Yes		Yes
dopaminergic synapse	3.84×10^{-11}			
toll like receptor signaling pathway	1.18×10^{-10}	Yes		Yes
tnf signaling pathway	1.50×10^{-10}			
glutamatergic synapse	1.67×10^{-10}			
ampk signaling pathway	4.93×10^{-10}			
osteoclast differentiation	6.68×10^{-10}			
apoptosis	1.13×10^{-09}	Yes		Yes
gap junction	1.32×10^{-07}	Yes		Yes
cell cycle	2.99×10^{-07}	Yes		Yes
oocyte meiosis	4.41×10^{-07}			
circadian entrainment	1.18×10^{-05}			
long term potentiation	1.63×10^{-05}			

vascular smooth muscle contraction	5.50×10^{-05}			
wnt signaling pathway	7.60×10^{-05}			
glycolysis gluconeogenesis	0.5000		Yes	
tyrosine metabolism	0.5000		Yes	
beta alanine metabolism	0.5000		Yes	
histidine metabolism	0.5000		Yes	
arginine and proline metabolism	0.5000		Yes	
glycine serine and threonine metabolism	0.5000		Yes	
platinum drug resistance	0.5000			
hif 1 signaling pathway	0.5000			
tryptophan metabolism	0.5000		Yes	
hippo signaling pathway	0.5000			
tight junction	0.5000	Yes		
retrograde endocannabinoid signaling	0.5000			
pyrimidine metabolism	0.5001		Yes	
purine metabolism	0.5002		Yes	
calcium signaling pathway	0.5004	Yes		Yes
mapk signaling pathway	0.5010	Yes		Yes
starch and sucrose metabolism	1		Yes	
insulin secretion	1			
melanogenesis	1			Yes
ovarian steroidogenesis	1			
thyroid hormone synthesis	1			
aldosterone synthesis and secretion	1			
glucagon signaling pathway	1			
arachidonic acid metabolism	1		Yes	
p53 signaling pathway	1	Yes		
mrna surveillance pathway	1			
protein processing in endoplasmic reticulum	1			
apoptosis multiple species	1			
nod like receptor signaling pathway	1			
transcriptional misregulation in cancer	1			
glutathione metabolism	1		Yes	
gabaergic synapse	1			
renin secretion	1			
salivary secretion	1			
gastric acid secretion	1			
pancreatic secretion	1			
dorso ventral axis formation	1			
th17 cell differentiation	1			
adipocytokine signaling pathway	1	Yes		Yes
endocrine and other factor regulated calcium reabsorption	1			
vasopressin regulated water reabsorption	1			
cytokine cytokine receptor interaction	1			Yes
endocytosis	1			
adherens junction	1	Yes		Yes
fatty acid degradation	1		Yes	
fatty acid metabolism	1			
citrate cycle tca cycle	1		Yes	
pyruvate metabolism	1		Yes	
proximal tubule bicarbonate reclamation	1			
carbon metabolism	1			
tgf beta signaling pathway	1			
cardiac muscle contraction	1			
lysine degradation	1		Yes	
ecm receptor interaction	1			Yes
pentose and glucuronate interconversions	1		Yes	
ascorbate and aldarate metabolism	1		Yes	
porphyrin and chlorophyll metabolism	1			
cytosolic dna sensing pathway	1			
neuroactive ligand receptor interaction	1			
taste transduction	1			
th1 and th2 cell differentiation	1			
olfactory transduction	1			
intestinal immune network for iga production	1			
antifolate resistance	1			
hematopoietic cell lineage	1			
cell adhesion molecules cams	1			
phototransduction	1			
glycerophospholipid metabolism	1		Yes	
ether lipid metabolism	1		Yes	
alpha linolenic acid metabolism	1			
glycerolipid metabolism	1		Yes	
primary bile acid biosynthesis	1			
alanine aspartate and glutamate metabolism	1		Yes	

butanoate metabolism	1		Yes
fatty acid biosynthesis	1		
peroxisome	1		
fat digestion and absorption	1		
rig i like receptor signaling pathway	1	Yes	
synthesis and degradation of ketone bodies	1		Yes
valine leucine and isoleucine degradation	1		Yes
terpenoid backbone biosynthesis	1		Yes
biosynthesis of unsaturated fatty acids	1		
phagosome	1	Yes	
propanoate metabolism	1		Yes
vitamin b6 metabolism	1		
nicotinate and nicotinamide metabolism	1		Yes
hippo signaling pathway multiple species	1		
thiamine metabolism	1		
one carbon pool by folate	1		Yes

Table S8. Results obtained by employing SubPathway-GM [1] to extract and enrich BRCA subpathways using KEGG pathway terms. The terms are sorted by means of p-value. We also reports if the other methods detect the same term as statistically significant ($p < 0.01$). In italics we report the top-20 pathways used for the comparisons.

**Pathway**	**SPECifIC**	**SubPathway-GM**	**SubPathway-GMir**	**DEsubs**
cell cycle	*Yes*	7.08×10^{-11}		*Yes*
focal adhesion	*Yes*	2.25×10^{-10}		*Yes*
complement and coagulation cascades		2.00×10^{-08}		
ppar signaling pathway	*Yes*	5.00×10^{-08}		*Yes*
tight junction		8.74×10^{-08}		
mapk signaling pathway		1.07×10^{-06}		*Yes*
p53 signaling pathway		1.07×10^{-06}		
neurotrophin signaling pathway	*Yes*	2.71×10^{-06}		
jak stat signaling pathway	*Yes*	1.69×10^{-05}		*Yes*
chemokine signaling pathway	*Yes*	6.46×10^{-05}		
regulation of actin cytoskeleton	*Yes*	7.65×10^{-05}		*Yes*
insulin signaling pathway	*Yes*	*0.0002*		
t cell receptor signaling pathway	*Yes*	*0.0004*		
fc gamma r mediated phagocytosis	*Yes*	*0.0007*		
calcium signaling pathway		*0.0008*		*Yes*
notch signaling pathway		*0.0016*		*Yes*
phagosome		*0.0041*		
glycosaminoglycan biosynthesis chondroitin sulfate dermatan sulfate		*0.0072*		
b cell receptor signaling pathway	*Yes*	*0.0078*		
gap junction	*Yes*	*0.0078*		*Yes*
adherens junction		0.0078		Yes
apoptosis	Yes	0.0096		Yes
toll like receptor signaling pathway	Yes	0.0099		Yes
adipocytokine signaling pathway		0.0099		Yes
rig i like receptor signaling pathway		0.0099		
vegf signaling pathway	Yes	0.0104		Yes
dorso ventral axis formation		0.0133		
gnrh signaling pathway	Yes	0.0185		Yes
phenylalanine metabolism	Yes	0.0207	Yes	
hedgehog signaling pathway		0.0407		Yes
glycerolipid metabolism		0.0408	Yes	
leukocyte transendothelial migration	Yes	0.0443		
ecm receptor interaction		0.0450		Yes
mtor signaling pathway	Yes	0.0458		
progesterone mediated oocyte maturation	Yes	0.0458		
tgf beta signaling pathway		0.0458		
glycosylphosphatidylinositol gpi anchor biosynthesis		0.0458		
vascular smooth muscle contraction	Yes	0.0506		
nod like receptor signaling pathway		0.0511		
natural killer cell mediated cytotoxicity	Yes	0.0540		Yes
axon guidance	Yes	0.0560		
n glycan biosynthesis		0.0560	Yes	
drug metabolism cytochrome p450	Yes	0.0619	Yes	
long term potentiation	Yes	0.0780		
tyrosine metabolism		0.0780	Yes	
circadian rhythm		0.0812		

pathways in cancer	Yes	0.0868		
pyruvate metabolism		0.0887	Yes	
wnt signaling pathway	Yes	0.1024		
aldosterone regulated sodium reabsorption	Yes	0.1028		
phosphatidylinositol signaling system	Yes	0.1162		
cell adhesion molecules cams		0.1162		
erbb signaling pathway	Yes	0.1454		
glycolysis gluconeogenesis		0.1572	Yes	
cysteine and methionine metabolism		0.1593	Yes	
butanoate metabolism		0.1615	Yes	
pancreatic secretion		0.1630		
pyrimidine metabolism		0.1671	Yes	
arginine and proline metabolism		0.1747	Yes	
mucin type o glycan biosynthesis		0.1870	Yes	
inositol phosphate metabolism	Yes	0.2146	Yes	
histidine metabolism		0.2146	Yes	
vasopressin regulated water reabsorption		0.2146		
long term depression	Yes	0.2267		Yes
beta alanine metabolism		0.2440	Yes	
glycerophospholipid metabolism		0.2440	Yes	
ether lipid metabolism		0.2440	Yes	
sphingolipid metabolism		0.2593	Yes	
galactose metabolism		0.2717	Yes	
amino sugar and nucleotide sugar metabolism		0.2721	Yes	
gastric acid secretion		0.2761		
glycine serine and threonine metabolism		0.2797	Yes	
glycosphingolipid biosynthesis globo and isoglobo series		0.2864		
salivary secretion		0.2947		
valine leucine and isoleucine degradation		0.2947	Yes	
fructose and mannose metabolism		0.3022	Yes	
taste transduction		0.3127		
purine metabolism		0.3660	Yes	
endocytosis		0.3660		
pantothenate and coa biosynthesis		0.4057		
drug metabolism other enzymes	Yes	0.4292	Yes	
caffeine metabolism		0.4307		
glycosphingolipid biosynthesis ganglio series		0.4307		
nitrogen metabolism		0.4307	Yes	
oocyte meiosis	Yes	0.4833		
melanogenesis		0.4870		Yes
fc epsilon ri signaling pathway	Yes	0.4894		Yes
propanoate metabolism		0.5207	Yes	
tryptophan metabolism		0.5248	Yes	
synthesis and degradation of ketone bodies		0.5248	Yes	
phenylalanine tyrosine and tryptophan biosynthesis		0.5248		
riboflavin metabolism		0.5248	Yes	
one carbon pool by folate		0.5316	Yes	
terpenoid backbone biosynthesis		0.5957	Yes	
cytosolic dna sensing pathway		0.6002		
alanine aspartate and glutamate metabolism		0.6023	Yes	
arachidonic acid metabolism		0.6041	Yes	
glycosaminoglycan degradation		0.6041	Yes	
citrate cycle tca cycle		0.6053	Yes	
glycosaminoglycan biosynthesis heparan sulfate heparin		0.6053		
glycosphingolipid biosynthesis lacto and neolacto series		0.6231	Yes	
selenocompound metabolism		0.6296	Yes	
protein processing in endoplasmic reticulum		0.6463		
pentose phosphate pathway		0.6463	Yes	
fatty acid biosynthesis		0.6569		
lysine degradation		0.6803	Yes	
glyoxylate and dicarboxylate metabolism		0.6936		
lysine biosynthesis		0.6936		
steroid biosynthesis		0.7141	Yes	
folate biosynthesis		0.7141	Yes	
primary bile acid biosynthesis		0.7374		
cytokine cytokine receptor interaction		0.7499		Yes
sulfur metabolism		0.8436		
fatty acid degradation		0.8545	Yes	
glutathione metabolism		0.8911	Yes	
nicotinate and nicotinamide metabolism		0.9075	Yes	
steroid hormone biosynthesis	Yes	0.9227	Yes	
vitamin b6 metabolism		0.9464		
starch and sucrose metabolism		0.9551	Yes	
antigen processing and presentation		0.9874		
pentose and glucuronate interconversions		1	Yes	

porphyrin and chlorophyll metabolism	1	
olfactory transduction	1	
fatty acid elongation	1	Yes

Table S9. Results obtained by employing SubPathway-GMir [2] to extract and enrich BRCA subpathways using KEGG pathway terms. The terms are sorted by means of p-value. We also reports if the other methods detect the same term as statistically significant ($p < 0.01$). In italics we report the top-20 pathways used for the comparisons.

Pathway	SPECifIC	SubPathway-GM	SubPathway-GMir	DEsubs
glycolysis gluconeogenesis			*0*	
pyrimidine metabolism			*0*	
purine metabolism			*0*	
glycerophospholipid metabolism			*0*	
fatty acid degradation			1.49×10^{-14}	
inositol phosphate metabolism	*Yes*		1.13×10^{-13}	
lysine degradation			3.73×10^{-13}	
pyruvate metabolism			1.93×10^{-12}	
valine leucine and isoleucine degradation			5.51×10^{-12}	
glycerolipid metabolism			6.46×10^{-12}	
tyrosine metabolism			2.42×10^{-11}	
n glycan biosynthesis			3.51×10^{-11}	
arachidonic acid metabolism			5.60×10^{-11}	
ether lipid metabolism			6.38×10^{-11}	
arginine and proline metabolism			9.10×10^{-10}	
amino sugar and nucleotide sugar metabolism			2.21×10^{-09}	
glutathione metabolism			2.41×10^{-09}	
sphingolipid metabolism			2.61×10^{-09}	
propanoate metabolism			1.15×10^{-08}	
glycosphingolipid biosynthesis lacto and neolacto series			1.15×10^{-08}	
galactose metabolism			1.40×10^{-08}	
fructose and mannose metabolism			1.40×10^{-08}	
phenylalanine metabolism	Yes		4.24×10^{-08}	
cysteine and methionine metabolism			5.24×10^{-08}	
drug metabolism other enzymes	Yes		1.01×10^{-07}	
alanine aspartate and glutamate metabolism			1.01×10^{-07}	
fatty acid elongation			1.21×10^{-07}	
starch and sucrose metabolism			1.37×10^{-07}	
butanoate metabolism			2.19×10^{-07}	
tryptophan metabolism			2.84×10^{-07}	
aminoacyl trna biosynthesis			5.87×10^{-07}	
metabolism of xenobiotics by cytochrome p450	Yes		1.02×10^{-06}	
steroid hormone biosynthesis	Yes		1.03×10^{-06}	
drug metabolism cytochrome p450	Yes		2.04×10^{-06}	
citrate cycle tca cycle			2.04×10^{-06}	
mucin type o glycan biosynthesis			2.04×10^{-06}	
pentose phosphate pathway			2.82×10^{-06}	
linoleic acid metabolism	Yes		7.36×10^{-06}	
glycine serine and threonine metabolism			7.49×10^{-06}	
histidine metabolism			2.10×10^{-05}	
pentose and glucuronate interconversions			0.0002	
steroid biosynthesis			0.0002	
terpenoid backbone biosynthesis			0.0002	
one carbon pool by folate			0.0002	
folate biosynthesis			0.0014	
ascorbate and aldarate metabolism			0.0019	
synthesis and degradation of ketone bodies			0.0019	
riboflavin metabolism			0.0019	
nicotinate and nicotinamide metabolism			0.0024	
glycosaminoglycan degradation			0.0057	
beta alanine metabolism			0.0058	
nitrogen metabolism			0.0058	
selenocompound metabolism			0.0058	
glycosphingolipid biosynthesis ganglio series			0.0188	

Table S10. Results obtained by employing DEsubs [3] to extract and enrich BRCA subpathways using KEGG pathway terms. The terms are sorted by means of p-value. We also reports if the other methods detect the same term as statistically significant ($p < 0.01$). In italics we report the top-20 pathways used for the comparisons.

Pathway	SPECifIC	SubPathway-GM	SubPathway-GMir	DEsubs
focal adhesion	*Yes*	*Yes*		*76*
cytokine cytokine receptor interaction				*59*
cell cycle	*Yes*	*Yes*		*56*
ecm receptor interaction				*30*
jak stat signaling pathway	*Yes*	*Yes*		*24*
ppar signaling pathway	*Yes*	*Yes*		*21*
mapk signaling pathway		*Yes*		*18*
toll like receptor signaling pathway	*Yes*	*Yes*		*14*
adherens junction		*Yes*		*13*
gnrh signaling pathway	*Yes*			*10*
long term depression	*Yes*			*10*
hedgehog signaling pathway				*10*
natural killer cell mediated cytotoxicity	*Yes*			*8*
regulation of actin cytoskeleton	*Yes*	*Yes*		*8*
notch signaling pathway		*Yes*		*8*
apoptosis	*Yes*	*Yes*		*6*
adipocytokine signaling pathway		*Yes*		*6*
vegf signaling pathway	*Yes*			*5*
fc epsilon ri signaling pathway	*Yes*			*5*
gap junction	*Yes*	*Yes*		*4*
calcium signaling pathway		Yes		4
melanogenesis				4

Table S11. p-Values, computed by means of Wilcoxon rank-sum test, for the comparison between the four methodologies in terms of distances from a disease node to a subpathway. The values where computed for the BRCA dataset.

	SPECifIC	Subpathay-GM	Subpathway-Gmir	DESubs
**SPECifIC**	-	0.0062	1.68×10^{-9}	3.98×10^{-5}
**Subpathay-GM**		-	2.98×10^{-6}	1.01×10^{-5}
**Subpathway-Gmir**			-	0.1794
**DESubs**				-

Table S12. p-Values, computed by means of Wilcoxon rank-sum test, for the comparison between the four methodologies in terms of distances from a disease node to a subpathway. The values where computed for the COAD dataset.

	SPECifIC	Subpathay-GM	Subpathway-Gmir	DESubs
**SPECifIC**	-	2.26×10^{-7}	8.32×10^{-12}	8.41×10^{-19}
**Subpathay-GM**		-	1.03×10^{-6}	2.44×10^{-13}
**Subpathway-Gmir**			-	4.08×10^{-07}
**DESubs**				-

Table S13. p-Values, computed by means of Wilcoxon rank-sum test, for the comparison between the four methodologies in terms of distances between disease nodes in a subpathway. The values where computed for the BRCA dataset. No p-values could be computed for DESubs since no disease nodes where found inside substructures.

	KEGG Pathways	SPECifIC	Subpathay-GM	Subpathway-Gmir	DESubs
**KEGG Pathways**	-	7.23×10^{-24}	0.0118	5.49×10^{-24}	-
**SPECifIC**		-	0.7974	4.56×10^{-11}	-
**Subpathay-GM**			-	6.50×10^{-6}	-
**Subpathway-Gmir**				-	-
**DESubs**					-

Table S14. p-Values, computed by means of Wilcoxon rank-sum test, for the comparison between the four methodologies in terms of distances between disease nodes in a subpathway. The values where computed for the COAD dataset. No p-values could be computed for DESubs since no disease nodes where found inside substructures.

	KEGG Pathways	SPECifIC	Subpathay-GM	Subpathway-Gmir	DESubs
**KEGG Pathways**	-	0.0155	1.74×10^{-5}	6.77×10^{-23}	-
**SPECifIC**		-	1.67×10^{-7}	6.51×10^{-33}	-
**Subpathay-GM**			-	4.05×10^{-5}	-
**Subpathway-Gmir**				-	-
**DESubs**					-

Bibliography

1. Li, C.; Han, J.; Yao, Q.; Zou, C.; Xu, Y.; Zhang, C.; Shang, D.; Zhou, L.; Zou, C.; Sun, Z.; others. Subpathway-GM: identification of metabolic subpathways via joint power of interesting genes and metabolites and their topologies within pathways. *Nucleic acids research* **2013**, *41*, e101–e101.
2. Feng, L.; Xu, Y.; Zhang, Y.; Sun, Z.; Han, J.; Zhang, C.; Yang, H.; Shang, D.; Su, F.; Shi, X.; others. Subpathway-GMir: identifying miRNA-mediated metabolic subpathways by integrating condition-specific genes, microRNAs, and pathway topologies. *Oncotarget* **2015**, *6*, 39151.
3. Vrahatis, A.G.; Balomenos, P.; Tsakalidis, A.K.; Bezerianos, A. DEsubs: an R package for flexible identification of differentially expressed subpathways using RNA-seq experiments. *Bioinformatics* **2016**, p. btw544.
4. Viglietto, G.; Motti, M.L.; Bruni, P.; Melillo, R.M.; D'Alessio, A.; Califano, D.; Vinci, F.; Chiappetta, G.; Tsichlis, P.; Bellacosa, A.; Fusco, A.; Santoro, M. Cytoplasmic relocalization and inhibition of the cyclin-dependent kinase inhibitor p27Kip1 by PKB/Akt-mediated phosphorylation in breast cancer. *Nature Medicine* **2002**, *8*, 1136–1144.
5. Jiang, P.; Enomoto, A.; Takahashi, M. Cell biology of the movement of breast cancer cells: Intracellular signalling and the actin cytoskeleton. *Cancer letters* **2009**, *284*, 122–130.
6. Hoover, K.B.; Liao, S.Y.; Bryant, P.J. Loss of the Tight Junction MAGUK ZO-1 in Breast Cancer. *The American Journal of Pathology* **1998**, *153*, 1767–1773.
7. Kominsky, S.L.; Argani, P.; Korz, D.; Evron, E.; Raman, V.; Garrett, E.; Rein, A.; Sauter, G.; Kallioniemi, O.P.; Sukumar, S. Loss of the tight junction protein claudin-7 correlates with histological grade in both ductal carcinoma in situ and invasive ductal carcinoma of the breast. *Oncogene* **2003**, *22*, 2021–2033.
8. WE, P.; AS, W.; JS, M.; D, R.; ER, F. Frequent alterations in E-cadherin and alpha- and beta-catenin expression in human breast cancer cell lines. *Oncogene* **1995**, *11*, 1319–1326.
9. McLachlan, E.; Shao, Q.; Laird, D.W. Connexins and Gap Junctions in Mammary Gland Development and Breast Cancer Progression. *Journal of Membrane Biology* **2007**, *218*, 107–121.

10. Farnie, G.; Clarke, R.B. Mammary Stem Cells and Breast Cancer—Role of Notch Signalling. *Stem cell reviews* **2007**, *3*, 169–175.
11. Takebe, N.; Warren, R.Q.; Ivy, S.P. Breast cancer growth and metastasis: interplay between cancer stem cells, embryonic signaling pathways and epithelial-to-mesenchymal transition. *Breast Cancer Research* **2011**, *13*, 211.
12. Mittal, S.; Subramanyam, D.; Dey, D.; Kumar, R.V.; Rangarajan, A. Cooperation of Notch and Ras/MAPK signaling pathways in human breast carcinogenesis. *Molecular Cancer* **2009**, *8*, 128.
13. Ebi, H.; Costa, C.; Faber, A.C.; Nishtala, M.; Kotani, H.; Juric, D.; Della Pelle, P.; Song, Y.; Yano, S.; Mino-Kenudson, M.; Benes, C.H.; Engelman, J.A. PI3K regulates MEK/ERK signaling in breast cancer via the Rac-GEF, P-Rex1. *Proceedings of the National Academy of Sciences* **2013**, *110*, 21124–21129.
14. Shekhar, M.P.; Pauley, R.; Heppner, G. Host microenvironment in breast cancer development: Extracellular matrix–stromal cell contribution to neoplastic phenotype of epithelial cells in the breast. *Breast Cancer Research* **2003**, *5*, 130.
15. Emons, G.; ºndker, C.G.; ºnthert, A.R.G.; Westphalen, S.; Kavanagh, J.; Verschraegen, C. GnRH antagonists in the treatment of gynecological and breast cancers. *Endocrine-Related Cancer* **2003**, *10*, 291–299.
16. McMahon, G. VEGF Receptor Signaling in Tumor Angiogenesis. *The Oncologist* **2000**, *5*, 3–10.
17. Simpson, E.R.; Brown, K.A. Minireview: Obesity and Breast Cancer: A Tale of Inflammation and Dysregulated Metabolism. *Molecular endocrinology* **2013**, *27*, 715–725.
18. Dean, S.J.; Rhodes, A. Triple negative breast cancer: the role of metabolic pathways. *Malays J Pathol* **2014**.
19. Sarraf, P.; Mueller, E.; Jones, D.; King, F.J.; DeAngelo, D.J.; Partridge, J.B.; Holden, S.A.; Chen, L.B.; Singer, S.; Fletcher, C.; Spiegelman, B.M. Differentiation and reversal of malignant changes in colon cancer through PPAR|[ggr]|. *Nature Medicine* **1998**, *4*, 1046–1052.
20. Michalik, L.; Wahli, W. PPARs Mediate Lipid Signaling in Inflammation and Cancer. *PPAR research* **2008**, *2008*, 1–15.
21. Hollande, F.; Papin, M. Tight Junctions in Colorectal Cancer. In *Tight Junctions in Cancer Metastasis*; Springer Netherlands: Dordrecht, 2013; pp. 149–167.
22. Albasri, A.; Fadhil, W.; Scholefield, J.H.; Durrant, L.G.; Ilyas, M. Nuclear expression of phosphorylated focal adhesion kinase is associated with poor prognosis in human colorectal cancer. *Anticancer research* **2014**, *34*, 3969–3974.
23. Watson, A.J.M. Apoptosis and colorectal cancer. *Gut* **2004**, *53*, 1701–1709.
24. Fang, J.Y.; Richardson, B.C. The MAPK signalling pathways and colorectal cancer. *The lancet oncology* **2005**, *6*, 322–327.
25. Vermeulen, L.; De Sousa E Melo, F.; van der Heijden, M.; Cameron, K.; de Jong, J.H.; Borovski, T.; Tuynman, J.B.; Todaro, M.; Merz, C.; Rodermond, H.; Sprick, M.R.; Kemper, K.; Richel, D.J.; Stassi, G.; Medema, J.P. Wnt activity defines colon cancer stem cells and is regulated by the microenvironment. *Nature Cell Biology* **2010**, *12*, 468–476.
26. Manna, S.K.; Tanaka, N.; Krausz, K.W.; Haznadar, M.; Xue, X.; Matsubara, T.; Bowman, E.D.; Fearon, E.R.; Harris, C.C.; Shah, Y.M.; Gonzalez, F.J. Biomarkers of Coordinate Metabolic Reprogramming in Colorectal Tumors in Mice and Humans. *Gastroenterology* **2014**, *146*, 1313–1324.
27. Hirayama, A.; Kami, K.; Sugimoto, M.; Sugawara, M.; Toki, N.; Onozuka, H.; Kinoshita, T.; Saito, N.; Ochiai, A.; Tomita, M.; Esumi, H.; Soga, T. Quantitative Metabolome Profiling of Colon and Stomach Cancer Microenvironment by Capillary Electrophoresis Time-of-Flight Mass Spectrometry. *Cancer Research* **2009**, *69*, 4918–4925.
28. Wang, H.; Tso, V.K.; Slupsky, C.M.; Fedorak, R.N. Metabolomics and detection of colorectal cancer in humans: a systematic review. *Future Oncology* **2010**, *6*, 1395–1406.
29. Chen, L.C.; Tu, S.H.; Huang, C.S.; Chen, C.S.; Ho, C.T.; Lin, H.W.; Lee, C.H.; Chang, H.W.; Chang, C.H.; Wu, C.H.; Lee, W.S.; Ho, Y.S. Human breast cancer cell metastasis is attenuated by lysyl oxidase inhibitors through down-regulation of focal adhesion kinase and the paxillin-signaling pathway. *Breast Cancer Research and Treatment* **2012**, *134*, 989–1004.
30. Vetvik, K.K.; Sonerud, T.; Lindeberg, M.; Lüders, T.; Størkson, R.H.; Jonsdottir, K.; Frengen, E.; Pietiläinen, K.H.; Bukholm, I. Globular adiponectin and its downstream target genes are up-regulated locally in human colorectal tumors: ex vivo and in vitro studies. *Metabolism* **2014**, *63*, 672–681.

31. Mazzarelli, P.; Pucci, S.; Bonanno, E.; Sesti, F.; Calvani, M.; Spagnoli, L.G. Carnitine palmitoyltransferase I in human carcinomas: A novel role in histone deacetylation? *Cancer Biology & Therapy* **2007**, *6*, 1606–1613.
32. Peng, Y.P.; Zhu, Y.; Zhang, J.J.; Xu, Z.K.; Qian, Z.Y.; Dai, C.C.; Jiang, K.R.; Wu, J.L.; Gao, W.T.; Li, Q.; Du, Q.; Miao, Y. Comprehensive analysis of the percentage of surface receptors and cytotoxic granules positive natural killer cells in patients with pancreatic cancer, gastric cancer, and colorectal cancer. *Journal of Translational Medicine* **2013**, *11*, 262.
33. Rocca, Y.S.; Roberti, M.P.; Arriaga, J.M.; Amat, M.; Bruno, L.; Pampena, M.B.; Huertas, E.; Loria, F.S.; Pairola, A.; Bianchini, M.; Mordoh, J.; Levy, E.M. Altered phenotype in peripheral blood and tumor-associated NK cells from colorectal cancer patients. *Innate Immunity* **2012**, *19*, 76–85.
34. Untersmayr, E.; Bises, G.; Starkl, P.; Bevins, C.L.; Scheiner, O.; Boltz-Nitulescu, G.; Wrba, F.; Jensen-Jarolim, E. The High Affinity IgE Receptor FcϵRI Is Expressed by Human Intestinal Epithelial Cells. *PLoS ONE* **2010**, *5*, e9023.
35. Francescone, R.; Hou, V.; Grivennikov, S.I. Cytokines, IBD and colitis-associated cancer. *Inflammatory bowel diseases* **2015**, *21*, 409–418.
36. Uchibori, R.; Tsukahara, T.; Mizuguchi, H.; Saga, Y.; Urabe, M.; Mizukami, H.; Kume, A.; Ozawa, K. NF-κB Activity Regulates Mesenchymal Stem Cell Accumulation at Tumor Sites. *Cancer Research* **2013**, *73*, 364–372.
37. Stepulak, A.; Rola, R.; Polberg, K.; Ikonomidou, C. Glutamate and its receptors in cancer. *Journal of Neural Transmission* **2014**, *121*, 933–944.
38. Piñero, J.; Queralt-Rosinach, N.; Bravo, À.; Deu-Pons, J.; Bauer-Mehren, A.; Baron, M.; Sanz, F.; Furlong, L.I. DisGeNET: a discovery platform for the dynamical exploration of human diseases and their genes. *Database* **2015**, *2015*, bav028.
39. Piñero, J.; Bravo, À.; Queralt-Rosinach, N.; Gutiérrez-Sacristán, A.; Deu-Pons, J.; Centeno, E.; García-García, J.; Sanz, F.; Furlong, L.I. DisGeNET: a comprehensive platform integrating information on human disease-associated genes and variants. *Nucleic Acids Research* **2016**, p. gkw943.
